# Difference of cardiac rehabilitation in the morning or evening on indexes of left ventricular and *N*-terminal pro-brain natriuretic peptide: a randomized controlled trial

**DOI:** 10.1097/MS9.0000000000000580

**Published:** 2023-06-09

**Authors:** Mostafa Dehghani, Mostafa Cheragi, Bahram Delfan, Morteza Dehghani, Amir Shakarami, Yagoob Bagheri, Parsa Namdari, Mehrdad Namdari

**Affiliations:** a Department of Cardiovascular research Center, Shahid Rahimi Hospital; b Department of Razi Herbal Medicines Research Center, Lorestan University of Medical Sciences, Khorramabad; c Shahid Beheshti University of Medical Sciences, Tehran, Iran; d Department of University of Debrecen, Debrecen, Faculty of Medicine, Hungary

**Keywords:** Cardiac Rehabilitation Morning or Evening, Echocardiographic Findings, Ventricular Functions, NT-proBNP

## Abstract

The present study aimed to evaluate the effects a cardiac rehabilitation program (CRP) performed in the morning or evening on left ventricular (LV) filling indices and the level of *N*-terminal fragment of pro-brain natriuretic peptide (NT-proBNP) in patients undergoing percutaneous coronary angioplasty during the COVID-19 pandemic.

**Methods:**

This was a randomized controlled single-blinded clinical trial. Ninety-six patients (mean age: 50.2 ± 8.1 years, 36 women and 44 men) with percutaneous coronary angioplasty were divided into two groups of intervention and control. In each group, the CRP was performed in either morning or evening. The CRP included walking and performing push-ups and sit-ups for 8 weeks. The participants of the control groups received routine care. The functional indices of LV, including LV ejection fraction, systolic function, and diastolic function (i.e. the transmitral flow), the E/e’ to left atrium peak strain ratio (as an estimation for LA stiffness), and NT-proBNP level were measured in all participants before starting and at the end of the CRP.

**Results:**

In the intervention group, the individuals performing the CRP in the evening had significantly higher E-wave (0.76±0.02 vs. 0.75±0.03; *P*=0.008), ejection fraction (52.5±5.64 vs. 55.5±3.59; *P*=0.011), and diastolic function velocity (E/A ratio, 1.03±0.06 vs. 1.05±0.03; *P*=0.014) and significantly lower A-wave (0.72±0.02 vs. 0.71±0.01; *P*=0.041), E/e’ ratio (6.74±0.29 vs. 6.51±0.38; *P*=0.038), and NT-proBNP level (2007.9±214.24 vs. 1933.9±253.13; *P*=0.045) compared with those performing the program in the morning.

**Conclusions:**

A supervised CRP performed in the evening compared with morning was more effective in improving LV functional indices. Therefore, such home-based interventions are recommended to be performed in the evening during the COVID-19 pandemic.

## Introduction

Highlights

- During the coronavirus pandemic, there were limitations in implementing cardiac rehabilitation programs in clinics; so, there is a crucial need for alternative cardiac rehabilitation program (CRP) approaches.
- The implementation an CRP-supervised telerehabilitation program significantly improved left ventricular systolic and diastolic functions in percutaneous coronary angioplasty patients during the COVID-19 pandemic.
- A challenging issue about time of day a cardia rehabilitation programs is sudden cardiac death even in healthy individuals.
- Performing the CRP in the evening more effectively improved left ventricular dysfunctions than in the morning.

Cardiovascular diseases (CVDs) are among the most dreadful health conditions and the primary causes of death and disability worldwide^1^. The best way to cope with this disease is to identify and effectively modify its main risk factors^2^.

During myocardial remodelling and cardiac repair, the levels of the *N*-terminal pro-brain natriuretic peptide (NT-proBNP) have been noted to be associated with the severity of myocardial ischaemia^3^ and left ventricular (LV) dysfunction^4^. Therefore, NT-proBNP is often used as a marker for predicting heart failure in patients with acute coronary syndrome^5^. Studies have suggested that cardiac rehabilitation programs (CRP) can alter NT-proBNP levels in patients with CVDs^6^.

The rapid spread of COVID-19 led to the cancellations of many medical visits and the temporary deferral of non-urgent selective medical procedures, including CRPs, due to decreased accessibility to on-site services. One way to prevent the shutdown of cardiac rehabilitation services during the COVID-19 pandemic is to develop home-based methods^6^. The use of telemedicine equipment, such as smart wristbands, can help implement home-based cardiac telerehabilitation (HBCT) programs. Remote, controlled, and supervised programs at the patient’s home can reduce the risk of COVID -19 transmission^7^.

Myocardial infarction (MI) often occurs in the early hours of the morning, which may be due to an increase in the sympathetic tone at this time, leading to the elevation of blood pressure, sympathetic nerve activity, heart rate, coronary artery contraction, and, consequently, myocardial load^8^. Although the health benefits of exercise activities have been known for a long time, investigations have indicated that exercise at certain day-night times can lead to sudden cardiac death (SCD) even in healthy persons^9^. This event is particularly accentuated in patients with CADs.

In this randomized clinical trial, the impact of the circadian rhythm was investigated on the effectiveness of a CRP, performed either in the morning or evening, in patients undergoing percutaneous coronary angioplasty (PCA) during the COVID-19 pandemic. We also reviewed the literature investigating the applicability of telemedicine in implementing CRPs (i.e. telerehabilitation programs) to encourage physicians and families to actively participate in home-based rehabilitation programs to improve patients’ LV functional parameters.

## Methods

### Participants

**Figure 1:**
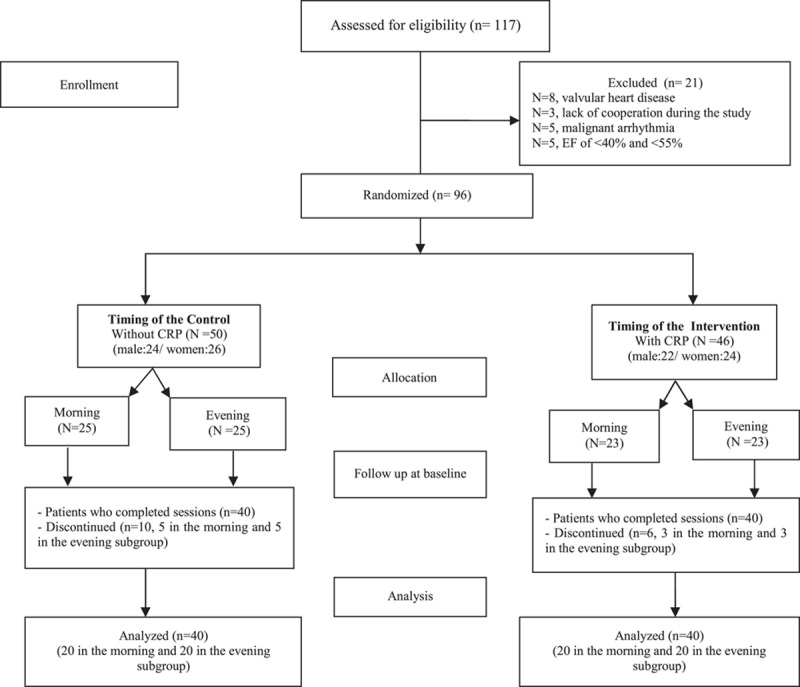
CONSORT flowchart of the randomised controlled trials. CRP, cardiac rehabilitation program.

This was a randomized single-blinded controlled trial conducted in line with CONSORT criteria (Fig. 1). Our aim was to examine the effects of an 8-week CRP (performed in either morning or evening) on LV function in patients with CVDs following PCA. For this, the patients were initially examined to determine their anthropometric specifications and BMI [MA801 Professional Body Composition Analyzer]. Each participant sat calmly for 10 min, and then systolic and diastolic blood pressures at rest were recorded (Beurer BM-16 Blood Pressure Monitor).

All the patients included in this study underwent PCA within 2 months after being discharged from the hospital. The patients admitted to the ICU of the cardiology ward of the Cardiovascular Research Center discharged after PCA (International Classification of Diseases, ICD-10 code: 125.7)^10^ from January to April 2020 were enroled in this study. All the methods employed in this research were registered at the Iranian Registry for Clinical Trials under the registration number of IRCT20181122041725N3. To control the effects of potentially confounding factors, only patients with the following inclusion criteria were recruited: history of angioplasty and/or angiography, relapse at least one month after MI or two months after complete revascularization PCA (with the culprit vessel-only approach), ejection fraction (EF) between 40 and 55%, the diagnosis of moderate cardiovascular disorder (New York Heart Association classes III and IV)^11,^ the lack of regular exercise activity during 3 months prior to the intervention, and no history of using a pacemaker. Patients with heart failure with preserved EF, heart failare with reduced EF, recurrent chest pain, conduction disturbances, valvular heart disease, and atrial ventricular malignant arrhythmias were excluded. Also, the patients attending less than half of the rehabilitation sessions and those with aspiration thrombectomy or under treatment with intravenous glycoprotein IIb/IIIa antagonists were excluded from the study. The study was approved by the Ethics Committee of Lorestan University of Medical Sciences (approval ID: IR.LUMS.REC.1399.200). Participants signed a written informed agreement before entering the study. The patients were selected among those undergoing drug-eluting stent placement within 100 min after admission and/or developing thrombolysis in the myocardial infarction flow after the procedure.

One hundred and seventeen patients were initially evaluated, of whom a total of 96 eligible individuals were allocated into the intervention and control groups via the permuted block randomization method. In each group, the participants were subdivided into two subgroups to perform the CRP either in the morning or evening. The patients who attended less than 50% of rehabilitation sessions were excluded from the study (*N*=21, Fig. 1).

### Study design

The CRP continued for 8 weeks (24 sessions), and testing was conducted at the baseline (before CRP) and post-intervention (after CRP). Before the start of the CRP, the patients attended two primary sessions: (1) to record their baseline data (left ventricular filling parameters and demographic characteristics), and (2) to become familiar with the study’s protocols and instructions. During the second meeting, the researcher also explained the correct form of performing CRP activities, including walking with smart wristbands and how to perform push-ups and sit-ups. The participants of the control group, either in the morning or evening subgroups, received routine care (i.e. unsupervised CRP for the same period). All exercises were monitored using a smart wristband (Xiaomi Mi Band 4)^12^. The patients were allowed to perform all the physical activities in the presence of the researcher to ensure they were okay with the smart wristbands and to familiarize themselves with the training. These two meetings were conducted 1 week prior to the first exercise session.

In this study, the independent variables included the CRP, the time of day, and COVID-19-area HBCT, as explained below. Dependent variables included LV filling indices (LV ejection fraction, systolic and diastolic functions) and the transmitral flow. Changes in these variables were compared between the groups before and after the CRP.

### CRP protocol

The CRP included twenty-four 50-min sessions (8 weeks, three sessions a week) and was implemented in either the morning (8:00–9:00 a.m.) or the evening (5:00–6:00 p.m.). The first 10 min included warm-up, followed by 40 min of aerobic walking on a flat surface while wearing the smart wristband, push-ups and sit-ups, and finally, 10-min cooling down. In the intervention group, exercises were monitored by a smart wristband^8,13^. From the week 2^nd^–8^th^, the number of steps, push-ups, and sit-ups gradually increased by 10% per week along with an increase in the load and intensity up to 70–85% of the heart rate^7,12^. A pilot study was conducted to standardize the protocol and specify the number of steps at the start of the study (4000 steps/day). The patients who agreed to participate provided a telephone number and designated a time appropriate for follow-up phone calls. The participants signed a written informed consent form before entering the study. Follow-up calls usually lasted four to 6 min and were made by a CRP physician twice weekly. All the patients of the intervention group also received weekly educations, including explanations about cardiovascular diseases, diagnostic and therapeutic approaches, their medications and complications, and their risk factors such as hypercholesterolaemia, smoking, obesity, physical inactivity, diabetes, obesity, hypertension, insufficient intake of fruits and vegetables, and psychosocial factors. Before the start of the CRP, a notebook containing the walking program was provided to the patients of the intervention group to record the number of their steps shown by the smart wristband. The patients were also requested to contact the researcher if they had any symptoms (e.g. chest pain, significant arrhythmias, etc.) during exercises, or if they had the intention to withdraw from the study. The subjects in the control group were advised by the physician to walk 40 min every other day (three sessions per week), but they did not have any supervised exercise plan during this period. During the study, all participants continued to receive their usual medications.

### Determining cardiovascular function and echocardiography parameters

**Figure 2:**
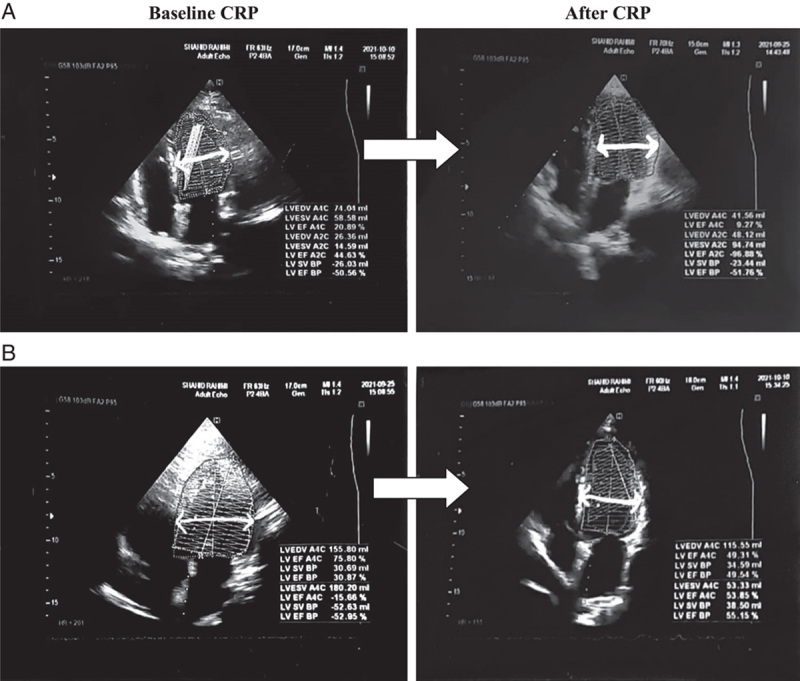
Ejection fraction calculation by Simpson’s method. Apical four-chamber view in systole (A4Cs) and diastole (A4Cd). (A) basline and after 8 weeks CRP in the morning experimental group. (B) basline and after 8 weeks CRP in the evening experimental group. CRP, cardiac rehabilitation program; EF, ejection fraction, LV, left ventricle.

As mentioned, the CRP protocol was implemented in a two-stage framework (i.e. before and after the 8-week CRP). Echocardiography uses sound waves to produce images to assess left ventricular function. LVEF-based parameters were evaluated clinically and by echocardiography using the Simpson’s method (Fig. 2). Echocardiographic measurements (General Electric, VIVID3) were performed before and after the 8-week CRP. The blood flow velocity at the mitral valve tip was regularly recorded using the pulsed wave Doppler echocardiography technique in four apical chambers. The peak early (E) and the peak late (A) waves were determined, and the E/A ratio was then measured. Left ventricle tissue velocity was measured by tissue Doppler imaging of the lateral mitral annulus (e’), and E/e’ was subsequently calculated. The ratio of E/e’ to left atrium (LA) peak strain was used to estimate LA stiffness.

### Determining NT-proBNP level

For determining NT-proBNP level, venous blood samples at fasting were collected from 08:00 to 09:00 in the morning. The patients were asked to avoid physical activity within 24 h before blood sampling. After a 12-h period, the tubes containing ethylenediamine tetra acetic acid were centrifuged at 5000 rpm for 15 min to separate plasma. Plasma samples were stored at −70 °C. In all patients, the level of NT-proBNP in the samples obtained before and after the CRP was determined using an ELISA kit (Roche Diagnostics).

### Randomization

First, we talked to the patients who had indications for undergoing rehabilitation about the advantages and disadvantages of home-based and in-hospital rehabilitation methods. Then the patients who were eligible for either method were randomized to the intervention and control groups using an allocation software through the block randomization method. In this method, the blocks were arranged randomly with letters A and B. We used blocks with sizes of 3, 6, and 4, so that the size of the tiles is the same. By combining the random blocks together, we created a balanced random list for the two treatment groups. Until the intended sample size in each group was reached, random allocation continued using the above blocks.

### Blinding

This study was a single-blinded trial, where the physician (researcher) and the patient were aware of the type of the rehabilitation program; however, the statistician who collected and analyzed the data did not know whether the patient was rehabilitated at a supervised or unsupervised method.

### Statistical methods

SPSS 25 (IBM Inc.) was used for statistical analysis. All continuous variables were expressed as mean ± SD, and categorical variables were expressed as number (n) and percentage (%). The paired *t*-test and χ^2^ test were used to determine significant differences in variables between or within the intervention and control groups. *P* less than 0.05 was considered as the statistical significance level. The ANCOVA test was used to compare the answers between the morning and evening times in each of the control and intervention groups, adjusting baseline values for the morning in the control group and for the evening in the intervention group.

The sample size was determined according to a previous study^14,^ and using the formula designed for comparing the means of two independent populations. Considering a confidence interval of 95% (alpha error rate of 5%), the beta error of 20%, power of 80%, an allocation ratio of 1:1:1:1, and the requirement for four pairwise comparisons, the sample size in each group was calculated as *n* = 40.

( $Z\left(1-\beta \right)=0.84,\;Z\left(1-\frac{\alpha }{2}\right)=1.96,\;s1\;=\;s2=1.4,\;x2\;=\;8.5,\mathrm{and}x1=9.25).$


$$n=\frac{2(Z\left(1-\frac{\alpha }{2}\right)+Z{\;{\left(1-\beta )\right)}^{2\;}\times \;S}^{2}}{{\left(x1-x2\right)}^{2}}$$

## Results

All patients (*n*=117) were randomly registered in this clinical trial. The participants’ anthropometric and clinical characteristics have been shown in Table 1. Overall, 96 participants with PCA were randomized to the control (*n* = 50) and intervention (*n* = 46) groups, and then each group was subdivided into the morning and evening subgroups. Age, BMI, history of addiction to substances, smoking, alcohol drinking habits, hypertension, hyperlipidemia, the family histories of heart diseases and diabetes, the distribution of MI, non-ST-segment elevation myocardial infarction, and ST-segment elevation myocardial infarction were not significantly different between the study groups.

**Table 1:** Baseline characteristics of the subjects in this research.

	Morning(*N*=40 )			Evening(*N*=40 )		
Variables	Experimental group (*N*=20)	Control group (*N*=20)	*P* value^*^	Experimental group (*N*=20)	Control group (*N*=20)	*P* value^*^
Age (year)	51.4±7.97	51.1±7.86	0.611	50.8±8.09	48.1±8.25	0.342
BMI (Kg/m^2^)	25.17±1.68	25.23±1.79	0.296	23.66±1.58	24.6±1.20	0.118
Sex (men/women)	11/9	13/8		10/10	11/9	
Smoking, *N* (%)	2 (10)	2 (10)	0.653	1 (10)	—	0.156
Hypertension, *N* (%)	3 (15)	4 (20)	0.371	3 (20)	2 (10)	0.204
Hyperlipidemia, *N* (%)	2 (10)	1 (5)	0.360	3 (15)	2 (10)	0.412
Family history of heart disease, *N* (%)	3 (15)	3 (15)	0.211	3 (30)	2 (10)	0.131
Myocardial infarction, *N* (%)	20 (100)	20 (100)		20 (100)	20 (100)	—
Diabetes mellitus, *N* (%)	1 (5)	1 (5)	0.756	2 (20)	2 (10)	0.698
NSTEMI, *N* (%)	1 (5)	2 (10)	0.134	2 (20)	2 (10)	0.372
STEMI, *N* (%)	2 (10)	1 (5)	0.347	2 (20)	2 (10)	0.251

The values have been expressed as mean ±SD, number or percentage.

NSTEMI, non-ST-segment elevation myocardial infarction; STEMI, ST-segment elevation myocardial infarction.

*Significant difference at *P*≤0.05.

### Changes in LV and systolic functional indices

Table 2 shows the effects of the CRP on the subjects’ LV and systolic function. In the initial assessment, there were no statistically significant differences between the groups regarding the studied variables.

**Table 2:** The comparison of ventricular diastolic and systolic functional parameters before and after cardiac rehabilitation program performed in either the morning or evening by patients with percutaneous coronary angioplasty.

			Control groups			Experimental groups				
Variables			Baseline (M ± SD)	After 8-week CRP (M ± SD)	*P* ^a^	Baseline (M ± SD)	After 8- week CRP (M ± SD)	*P* ^b^	*P* ^c^	*P* ^d^
E (m/s)	Time	Morning	0.75±0.02	0.74±0.03	0.905	0.74±0.02	0.75±0.03	0.046^*^	0.077	0.008^**^
		Evening	0.76±0.02	0.76±0.03	0.808	0.74±0.02	0.76±0.02	0.019^*^		
A (m/s)	Time	Morning	0.75±0.02	0.74±0.02	0.601	0.74±0.01	0.72±0.02	0.011^*^	0.195	0.041^*^
		Evening	0.76±0.01	0.75±0.02	0.240	0.75±0.01	0.71±0.01	0.008^**^		
E/A ratio	Time	Morning	0.99±0.02	0.99±0.05	0.180	0.99±0.03	1.03±0.06	0.021^*^	0.068	0.014^**^
		Evening	1.00±0.03	1.01±0.02	0.170	0.98±0.02	1.05±0.03	0.009^**^		
E/e’ ratio	Time	Morning	7.00±0.28	6.90±0.32	0.106	6.93±0.24	6.74±0.29	0.032^*^	0.475	0.038^*^
		Evening	6.94±0.24	6.82±0.32	0.076	6.91±0.35	6.51±0.38	0.003^**^		
LVEF %	Time	Morning	51.75±4.37	52.25±5.38	0.85	50±5.61	52.5±5.64	0.030^*^	0.156	0.011*
		Evening	49.75±5.09	50.00±4.86	0.170	50.75±4.66	55.5±3.59	<0.001^***^		
NT-proBNP(pg/ml)	Time	Morning	2092.2±113.4	2060.35±133.3	0.104	2104.5±142.64	2007.9±214.24	0.043	0.697	0.045*
		Evening	2104±127.24	2075.5±121.15	0.081	2094.65±136.29	1933.9±253.13	0.025		

Values expressed as mean ±SD, number or percentage. *P* ^a^ = *P* value control groups compared, and *P* ^b^ = *P* value experimental groups compared.; using paired *t*-test between before and after CRP. *P* ^c^ = *P* value compared of morning and evening control groups after 8 weeks CRP, and *P* ^d^ = *P* value compared of morning and evening experimental groups after 8 weeks CRP; using ANCOVA with baseline variable adjusted.

A, peak velocity of late wave; CRP, cardiac rehabilition program; E, Peak velocity of early wave; e’, early mitral annular tissue; LVEF, left ventricular ejection fraction; NT-proBNP, *N*-terminal fragment of pro-brain natriuretic peptide.

Significant difference before and after CRP performed either in the morning or evening. Significant difference *P*≤0.05.

*P<0.05,

**P<0.01,

***P<0.001.

Regarding within-group comparisons, significant differences were observed comparing LV diastolic and systolic functional parameters, including significant decreases in A-wave between morning experimental group (from 0.74±0.01 to 0.72±0.02 m/s, *P*=0.011), E/e’ (from 6.93±0.24 to 6.74±0.29 ratio; *P*=0.038) and between evening experimental group (from 0.75±0.01 to 0.71±0.01 m/s, *P*=0.008), E/e’ (from 6.91±0.35 to 6.51±0.38 ratio; *P*=0.003), and NT-proBNP level parameter morning experimental group (from 2104.5±142.64 to 2007.9±214.24 pg/ml, *P*=0.043), and between evening experimental group (from 2094.65±136.29 to 1933.9±253.13 pg/ml, *P*=0.025), also, significant increases in E-wave between morning experimental group (from 0.74±0.02 to 0.75±0.03 m/s, *P*=0.046), and E/A between morning experimental group (from 0.99±0.03 to 1.03±0.06 ratio, *P*=0.021), and between evening experimental group (from 0.98±0.02 to 1.05±0.03 ratio, *P*=0.009), and EF between morning experimental group (from 50±5.61 to 52.5±5.64%, *P*=0.030), and between evening experimental group (from 50.75±4.66 to 55.5±3.59%, *P*<0.001), in the experimental groups before and after the 8-week CRP (Table 2).

**Figure 3:**
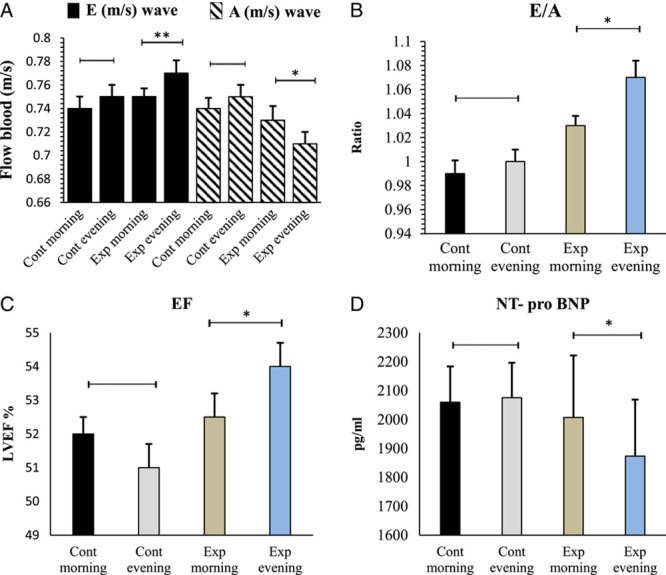
Comparing the effects of cardiac rehabilitation program timing on the E and A velocities, E/A, and LVEF in patients with percutaneous coronary angioplasty in the control (Cont) and experimental (Exp) groups and the morning and evening subgroups. A, peak velocity of late wave; E, peak velocity of early wave; LVEF, left ventricular ejection fraction; NT-proBNP; *N*-terminal fragment of pro-brain natriuretic peptide

After the 8-week CRP, significant differences were observed comparing LV diastolic and systolic functional parameters between the morning and evening experimental groups, including significantly lower A-wave (0.72±0.02 vs. 0.71±0.01; *P*=0.041), E/e’ (6.74±0.29 vs. 6.51±0.38; *P*=0.038), NT-proBNP level parameter (2007.9±214.24 vs. 1933.9±253.13; *P*=0.045) and significantly higher E-wave (0.76±0.02 vs. 0.75±0.03; *P*=0.008), E/A (1.03±0.06 vs. 1.05±0.03; *P*=0.014), EF (52.5±5.64 vs. 55.5±3.59; *P*=0.011) and (Table 2, Fig. 2 & 3). No significant differences were observed in the mentioned LV diastolic and systolic functional parameters in the control group before and after the 8-week CRP.

## Discussion

This randomized controlled trial was performed to compare the effects of a home-based supervised cardiac rehabilitation program performed either in the morning or evening on LV filling and LV remodelling parameters in the patients undergoing coronary angioplasty. The findings of this study showed that performing a CRP for 8 weeks in the evening vs. morning was more effective in improving LV systolic and diastolic functional indices (E and A velocities, E/A and E/e’ ratio, and EF). We also observed a significant reduction in the circulating concentrations of NT-proBNP in participants with moderate diastolic dysfunction. None of these parameters showed significant alternations in the control group.

While the COVID-19 pandemic continues to spread without a clear hope of its ending, individuals living with CADs need to ensure about receiving necessary health interventions to maintain their health and recovery. Due to the time-dependent variations in physiological parameters, a challenging issue about CRPs is to choose the right time for performing these activities. The COVID-19 pandemic has hurdled the delivery of in-hospital CRPs, highlighting the need for using alternative methods such as supervised telerehabilitation programs, particularly for high-risk patients. So, we need to improvise new methods and redesign existing strategies to meet the challenge of effective delivery of CRPs during this critical period. In this regard, HBCT can be used as a practical alternative to fill this gap^15–17^.

In previous studies, the clinical significance of circadian variations has been highlighted in the onset of chest pain, non-Q-wave acute myocardial infarction (AMI), and SCD^18^. The incidence of chest pain^19^ and myocardial infarction^20^ peaks around 6 a.m. to noon. Another study also showed that SCD (with a peak incidence between 7 a.m. and 9 a.m.) could precede with remarkable alterations in circadian levels. On the other hand, myocardial infarction occurs in about one-third of all SCD cases^21^. It is unknown if training in the morning compared to other times of the day can modulate the risk of cardiovascular events. In one study^22,^ patients with established CVDs who performed exercise either in the morning or evening showed comparable ventricular functional parameters.

Diastolic dysfunction can reduce cardiac output and exercise capacity^23,^ and E-wave velocity and E/e’ ratio are the most essential indicators of diastolic function used in clinical research^24^. Although LV systolic dysfunction independently predicts mortality in coronary artery diseases patients, there is little information on the effects of the time of performing physical activities on LVEF^25^. In patients with AMI and LV systolic and diastolic dysfunctions, physical activity-based CRP has significantly improved LVEF^26^. Another research showed that a 4-week home-based CRP program could improve LV function in AMI participants^27^. Aging, alcohol consumption, tachycardia, and hypertension are among the risk factors reducing the E velocity, while regular exercise, high ventricular compliance, and high workload can increase this parameter^28^. According to another study^29,^ evening physical activity had better protective effects than morning exercise against cardiovascular events. Similar to our observation, other studies have found that CVD patients walking in the evening had better improvements in cardiovascular functional indices compared to those who performed physical activity in the morning^30^.

In our study, the CRP was performed either in the morning (8:00–9:00 a.m.) or evening (5:00–6:00 p.m.). Our results showed that PCA patients who performed the program in the evening had a lower risk for expanded coronary artery occlusion compared with those exercising in the morning. Also, the E/A ratio, the E to early diastolic mitral annular tissue velocity (E/e’) ratio, and LVEF significantly improved in the patients performing the CRP in the evening compared with those doing morning exercises. The direct mechanisms underlying the variable effects of exercise daytime on coronary artery occlusion are unclear. Prolonged physical activities (such as walking) have been suggested to lower blood pressure and decrease coronary spasm (due to myocardial ischaemia), and in this way, reduce coronary artery wall stress and stenosis.

Left atrium (LA) size or volume is a powerful predictor of cardiovascular outcomes and can be used as a biomarker in various cardiac diseases^31^. In this study, echocardiographic evaluation asserted that the CRP significantly improved LA reconstruction and LV diastolic function, as evidenced by measuring E/e’. In another study, a 3-week moderate-intensity CRP conducted in the early post-MI period was proven to promote enhanced anti-inflammatory effects^32^ and to reduce systemic inflammation, preventing deleterious ventricular remodelling. Moreover, CRP has been noted to significantly mitigate metabolic risk factors^33^. Beside its benefits in terms of inflammatory and metabolic profiles, CRPs have been noted to prevent the progression of LV systolic impairment^34,35^ and to increase myocardial perfusion independent of coronary lesions^36^. Other mechanisms proposed include an increase in the uptake of calcium ions by the calcium pumps of the sarcoplasmic reticulum during the first diastolic phase, extending the myocardial resting period, and elevating the E velocity and E/A ratio^37^.

The direct mechanisms underlying the protective effects of evening-time exercises against coronary artery occlusion are unknown. Prior studies have shown that systolic and diastolic blood pressures are the highest at 8–10 a.m^38^. In addition, shear stress tends to elevate after morning physical activity^39,^ and the tone of the tremedous coronary artery is higher in the morning than evening, while its diameter is smaller in the early morning than in the evening^30^. Jones *et al.* ^38^ reported that flow-mediated vasodilation in patients with different forms of angina reduced in the early morning (6 a.m.) and improved by afternoon (2 p.m.) and evening (8 p.m.). Coronary spasms occur round the clock, peaking from the middle of the night to the early morning, and they may also be triggered by exercise during the day^40^. Morikawa *et al*.^40^ described that aerobic exercise in the evening reduced the incidence of cardiac attacks and risk of sudden death and improved vascular endothelial function, blood pressure, oxidative stress, and inflammation in patients with coronary angina.

In line with our study, the level of the natriuretic peptide, which more rapidly responds to various loading conditions of the ventricles, was shown to reduce following a CRP, which was associated with improved LV function and remodelling^4^. A causal relationship has been proposed between changes in NT-proBNP and LV filling and remodelling biomarkers. It seems that improved LV systolic and diastolic functional indices partly result from peripheral adaptations and reduction in NT-proBNP levels, and these outcomes are assumed to alleviate diastolic wall stress and LV remodelling.

Overall, our findings highlight the importance of HBCT along with supervised exercises in improving LV systolic and diastolic function and LVEF. We observed that evening-time CRP had better protective effects than morning-time activities against cardiovascular events and coronary spasms.

### Limitations

There are a number of noteworthy limitations in our study. First, the generalization of our results to outpatient clinics may be restricted because here patients were strictly observed for certain eligibility criteria and were followed up more carefully to ensure their compliance with the program, a condition that may be hard to achieve in reality and in clinics. Second, LV diastolic filling parameters, evaluated by transmitral Echo-Doppler, could be influenced by a variety of factors, for example, LV filling pressure, valvular insufficiency, ventricular diastolic function, valve diseases (stenosis and/or regurgitation), and viscoelastic properties of the myocardium. To control these parameters, all of our patients were managed to receive the same medications throughout the study. Besides, only patients with mild degrees of valve diseases were included in this study.

## Conclusions

During the COVID-19 pandemic, through careful collaborative and innovative planning, healthcare providers can exploit new methodologies and redesign existing strategies to meet the current challenge of delivering CRPs. The present study showed that a supervised home-based CRP could effectively reduce the incidence of coronary artery occlusion. Also, we observed that evening-time compared with morning-time CRP better preserved left ventricular functional parameters in patients undergoing PCA.

## Ethical approval

The present study was approved by the Ethics committee of Lorestan University of Medical Sciences (approval ID: IR.LUMS.REC.1399.200). The protocols were conducted according to the guidelines of Helsinki Declaration.

## Consent

Written informed consent was obtained from the patients.

## Source of funding

NA.

## Conflicts of interest disclosure

N/a.

## Research registration unique identifying number (UIN)

Registry URL: http://www.irct.ir. Iranian registry of clinical trial number: IRCT20181122041725N3.

## Provenance and peer review

Not commissioned, externally peer-reviewed.
